# Ultrasound-guided 10% dextrose interfascial hydrodissection for patients with myofascial pain syndrome: A retrospective observational study

**DOI:** 10.1097/MD.0000000000042587

**Published:** 2025-06-20

**Authors:** Tao Wu, Bao Ru, Haixin Song, Zhiping Liao, Xiaotian Yang, Fangchao Wu, Jianhua Li

**Affiliations:** a Department of Rehabilitation Medicine, National Regional Medical Center, Sir Run Run Shaw Hospital, Alaer Hospital, Zhejiang University School of Medicine, Alar City, Xinjiang, PR China; b Department of Rehabilitation Medicine, Sir Run Run Shaw Hospital, College of Medicine, Zhejiang University, Hangzhou, Zhejiang Province, PR China.

**Keywords:** dextrose, fascia, injections, myofascial pain syndromes, ultrasonography

## Abstract

Myofascial pain syndrome (MPS) is a prevalent condition that accounts for a significant proportion of musculoskeletal pain cases. Interfascial hydrodissection (IH) represents an innovative therapeutic intervention for MPS, particularly for those patients who have not found relief through conventional treatment methods. We conducted this study to evaluate the efficacy of the ultrasound-guided IH technique with 10% dextrose solution (D10W) for treating MPS. A retrospective analysis of clinical data was conducted from MPS patients who did not respond to conventional treatments. These patients subsequently received ultrasound-guided injections of D10W. The efficacy of this treatment modality was meticulously evaluated by quantitatively measuring the reduction in pain intensity in patients across 3 distinct posttreatment conditions: at rest, at nocturnal, and during exercise. Numerical Rating Scale and EQ-5D-5L were assessed at baseline, and at 4 and 12 weeks following the final injection. The pain scores, assessed 4 and 12 weeks posttreatment, demonstrated a significant reduction compared to pretreatment levels across rest, nocturnal, and exercise states (*P* < .05). The EQ-5D-5L results indicated that there were no significant differences in the dimensions of mobility, self-care, and usual activities when comparing before and posttreatment (*P* > .05). Conversely, a significant improvement was observed in the dimensions of pain/discomfort and anxiety/depression posttreatment relative to baseline measures (*P* < .05). No serious adverse effects were observed. Ultrasound-guided D10W IH has demonstrated significant efficacy in improving pain and alleviating anxiety/depression in patients with refractory MPS. The outcomes are promising for individuals with MPS who have not experienced improvement with conventional medical interventions.

## 1. Introduction

Myofascial pain syndrome (MPS) is one of the most common causes of musculoskeletal pain. Although the manifestations of MPS are complex, its episodes and persistence are triggered by myofascial trigger points.^[1]^ Various physical therapies for MPS, including stretching exercises, transcutaneous electrical nerve stimulation, and extracorporeal shockwave therapy, have shown positive effects. Nonsteroidal anti-inflammatory drugs, muscle relaxants, anticonvulsants, antidepressants, and botulinum toxin type A have played significant roles in managing chronic symptoms.^[2]^ However, a number of patients continue to experience dissatisfaction with the outcomes of traditional treatment modalities.

Interfascial hydrodissection (IH), a novel therapeutic approach for MPS, has gained attention in recent years and is colloquially known as “Liquid Knife.”^[3]^ This technique involves blunt dissection of the fascial interstitial spaces, which are the interfacing areas between adjacent muscles. In patients with MPS, adhesions within these interstitial spaces can limit muscle contraction, potentially contributing to the pain and discomfort associated with the condition. By injecting anesthetic or saline solution into the fascial interstitial spaces, the technique enhances muscle gliding, and decreases muscle tension, thus alleviating MPS symptoms.^[4]^ The efficacy of 10% dextrose solution (D10W) in IH as a treatment for patients with MPS remains to be determined. Here, we introduce and describe ultrasound-guided D10W IH to safely and effectively treat MPS. The primary hypothesis of our study was that ultrasound guided D10W IH would reduce the severity of pain in patients with MPS in the short and long term.

## 2. Methods

### 2.1. Patient selection

All data was collected retrospectively from our Physical Medicine and Rehabilitation outpatient clinic. Patients diagnosed with MPS who did not respond to conventional treatments and subsequently underwent ultrasound-guided 10% dextrose IH between June 2022 and June 2024 were enrolled in this study. Prior to treatment, written informed consent was obtained from all participants. The experimental protocol was reviewed and approved by the Ethics Committee of Sir Run Run Shaw Hospital in accordance with ethical guidelines, including the protection of patient privacy and the confidentiality of data collected during the trial (Research No. 2022-0153).

Eligible candidates for ultrasound-guided 10% dextrose IH met the following inclusion criteria: (1) aged between 20 and 80 years, with no gender restrictions, (2) clear trigger points in the limbs or trunk, with MPS confirmed by Gerwin clinical diagnostic criteria,^[5]^ (3) did not respond to medical therapy, physical therapy, manual therapy, or other conservative treatments, and Numerical Rating Scale (NRS) score ≥ 5, (4) with duration of symptom onset at least 2 months. Patients with motor weakness, radiculopathy, bleeding disorders, neurological, cognitive, psychiatric, or rheumatological conditions, as well as those with cancer, or inflammatory processes were excluded.

Demographic variables, including age, gender, education and employment status, height, weight, and body mass index, were recorded. We recorded pain scores under 3 distinct conditions (rest, nocturnal, and exercise states) and EQ-5D-5L score before the injection, 4 and 12 weeks after the final injection.

### 2.2. Ultrasound-guided injection and evaluation protocol

**Figure 1.**
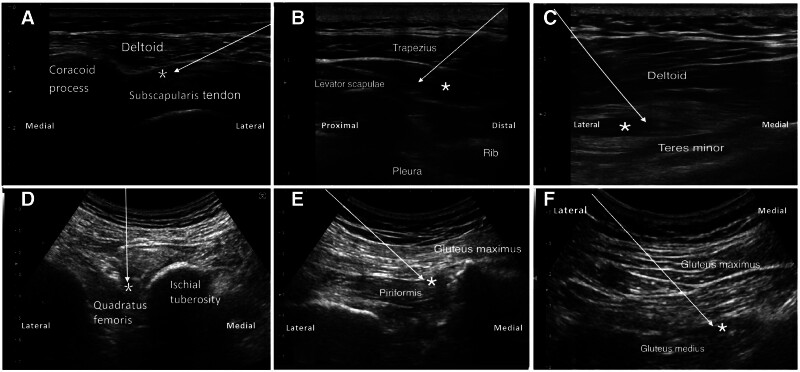
The procedure under real-time ultrasound guidance, where muscle twitching is observable upon needle tip contact with the lesion. Subsequently, a slow injection of a D10W is administered into the fascial space beneath the trigger point. The successful injection is indicated by the progressive separation of the fascial space as visualized with ultrasound guidance. The muscles involved are as follows: (A) subscapularis tendon, (B) levator scapulae, (C) teres minor, (D) quadratus femoris, (E) piriformis, (F) gluteus medius. The arrows represent the needle trajector, while the asterisk (*) marks the injectant.

Before the injection, a premixed solution with a concentration of 10% dextrose was prepared using a ratio of 50% dextrose 1 mL, 2% lidocaine 2 mL, and 0.9% sodium chloride 2 mL. Before the intervention, the physiatrist palpated the tender nodule to locate the injection muscle or tendon. To guide the intervention, an ultrasound machine (GE Logic Q) equipped with the linear or curvilinear array transducer (5–13 MHz) was used. The needle insertion site was aseptically prepared using povidone-iodine. With the use of in plane approach, the physiatrist injected the D10W into the fascia layer directly under the tender point with ultrasound guidance. Figure 1 shows the procedure scan of the IH. The ultrasound guided the needle tip to reach the target muscle fascial space, and muscle twitching was visible on the image when the needle tip contacted the trigger point. The myofascial trigger point appears as a hypoechoic nodule within the muscle tissue due to the presence of nociceptive mediators bound to water molecules and glycosaminoglycans.^[6]^ To ensure an accurate assessment of therapeutic efficacy, repeated needling on trigger points should be avoided to prevent the occurrence of the dry needle effect. Then, 5 mL 10% dextrose was slowly injected into each fascial space. The fascial layers appear as hyperechoic lines made of dense connective tissue separated by hypoechoic layers of loose connective tissue. The adhesions between the different layers can be considered the histological target of the ultrasound-guided hydrodissection to manage the myofascial pain.^[7]^ We separated the fascial space with the liquid, rather than the needle tip. The successful injection was indicated by the gradual separation of the fascial layer under ultrasound monitoring (see Fig. 1 & Supplemental Digital Content 1–4, Supplemental Digital Content, https://links.lww.com/MD/P195; https://links.lww.com/MD/P196; https://links.lww.com/MD/P197; https://links.lww.com/MD/P198). All patients received weekly outpatient follow-up assessments, and if the pain score was still > 3 (NRS), they received the second injection treatment. Patients received up to 3 injections, administered once a week. All patients were followed up for 12 weeks after the final injection treatment.

After the IH, all patients received education on fascial stretching exercises and were instructed to perform self-stretching exercises daily to consolidate the therapeutic effect of the injection.

### 2.3. Clinical efficacy assessment

NRS: NRS scores were obtained prior to the procedure, 4 and 12 weeks after the final injection. Severity of pain at rest, nocturnal, and exercise states was assessed. They were contacted through the outpatient clinic or by phone to gather the follow-up information. All data prior and after the procedure were taken by an assessor blinded to the intervention. The patients were asked to mark their perceived severity of pain on NRS graded between “0: no pain at all” and “10: worst pain imaginable.” NRS has been determined to be a reliable and valid scale for the patients with chronic pain. The NRS score ranges from 1 to 3 indicate mild pain, which is relatively tolerable and has little impact on daily life and sleep. Scores from 4 to 6 suggest moderate pain, which is more noticeable and may affect sleep but is still generally tolerable. Scores from 7 to 10 indicate that the pain is intense and unbearable, severely disrupting daily life and sleep. Patients may struggle to perform normal activities and may need medical intervention for relief. We have defined an NRS score of 3 or less as an indicator of effective treatment. Conversely, an NRS score >3 suggests dissatisfaction with the treatment outcomes.

EQ-5D-5L: The EQ-5D-5L is a health utility instrument comprising 5 dimensions: mobility, self-care, usual activities, pain/discomfort, and anxiety/depression.^[8]^ Each dimension is assessed with scores ranging from 1 to 5, where 1 represents the best (no problems) and 5 represents the worst (unable to perform/extreme difficulties). Depending on the participant’s preference, the Chinese version of the instrument was administered.

Adverse events: The study vigilantly monitored participants for acute bleeding as a primary immediate post-procedural complication. Additionally, the escalation of pain was assessed posttreatment as a secondary outcome measure. Participants were explicitly instructed to report any incidents of numbness or weakness in the regions that received the injection.

### 2.4. Statistical analysis

All data were entered into a custom-designed Excel spreadsheet. Data were analyzed using the SPSS Statistics 28.0 software program (IBM Corporation, Armonk). The homogeneity of variance was analyzed by Hartley test, Cochran–Cox test, and Bartlett chi-square test. NRS scores were presented as median and Interquartile Range (IQR). Related-samples Friedman 2-way analysis of variance by ranks was used to assess NRS score changes in measurements before and after the intervention. Descriptive statistics, including mean (standard deviations), and percentages, were calculated for EQ-5D-5L outcomes and adverse events, at multiple time points. A one-way ANOVA, along with post hoc independent T-tests, was conducted to assess EQ-5D-5L changes in outcome measures across these time points. The level of statistical significance was prespecified as *P* < .05.

## 3. Results

From June 2022 to June 2024, a total of 95 patients with refractory MPS were consecutively selected to receive 10% dextrose injections. Patients were on average 54.9 years (range: 18–80), 60 were men and 35 were women. The duration of symptom onset ranged from 75 to 185 days. The mean pain duration was 85.2 ± 26.6 days. The injection sites for the upper limb fascia included the supraspinatus (6 cases), infraspinatus (6 cases), teres minor (5 cases), deltoid (3 cases), and anconeus (4 cases). For the lower limb fascia treatment, injection sites included the gluteus medius (8 cases), gluteus minimus (4 cases), semitendinosus and semimembranosus (5 cases), biceps femoris (4 cases), and gastrocnemius (4 cases). The trunk fascia injection sites included the levator scapulae (15 cases), pectoralis minor (4 cases), trapezius (12 cases), rhomboid (8 cases), and erector spinae (7 cases).

Among the 95 individuals, 79 (83.1%) reported significant relief of symptoms following the initial treatment, indicating effective treatment (Pretreatment NRS ≥ 5; posttreatment NRS < 3). At the 12-week follow-up subsequent to the final treatment, 67 individuals (70.5%) continued to report satisfactory pain relief, with a NRS score of 3 or lower. 27 patients received 2 injections, and 38 patients received 3 injections at weekly intervals.

### 3.1. NRS evaluation

Pain scores recorded at rest, during the night, or exercise showed statistically significant decreases at the 4-week and 12-week follow-up in comparison to the baseline within the group (*P* < .05, refer to Table 1). However, no significant differences were observed in posttreatment pain levels among the groups assessed in restful, nocturnal, and active states at the 4-week and 12-week follow-up (*P* > .05, refer to Table 1).

**Table 1:** The severity of pain at rest, nocturnal, and during exercise states before interfascial hydrodissection, and 4 weeks, 12 weeks thereafter.

NRS	Median (IQR)				
	Before (n = 105)	4 weeks after (n = 105)	*P*	12 weeks after (n = 105)	*P*
At rest	5.0 (3.0)	2.0 (2.0)	<.05*	2.0 (2.0)	<.05**
At nocturnal	6.0 (3.0)	2.0 (2.0)	<.05*	2.0 (3.0)	<.05**
During exercise	6.0 (3.0)	2.0 (3.0)	<.05*	2.0 (3.0)	<.05**

IQR = interquartile range, NRS = numeric rating scale.

* *P* values between pretreatment and 4 week after treatment within the group;

** *P* value between pretreatment and 12 week after treatment within the group.

### 3.2. EQ-5D-5L evaluation

No significant differences were observed in the dimensions of mobility, self-care, and usual activities between pretreatment and posttreatment (*P* > .05, refer to Table 2). In contrast, there was a notable improvement in the dimensions of pain/discomfort and anxiety/depression following treatment as compared to the baseline measures (*P* < .05, refer to Table 2).

**Table 2:** Assessment of EQ-5D-5L before interfascial hydrodissection, and 4 weeks, 12 weeks thereafter.

	EQ-5D-5L measures (Mean ± SD)				
	Mobility	Self-care	Usual activities	Pain/discomfort	Anxiety/ Depression
Before	1.33 ± 0.73	1.46 ± 0.84	1.58 ± 0.69	3.17 ± 0.67	2.478 ± 0.95
4 weeks after	1.21 ± 0.44	1.35 ± 0.9	1.44 ± 0.55	1.66 ± 0.66	1.51 ± 0.57
12 weeks after	1.27 ± 0.6	1.38 ± 0.71	1.49 ± 0.73	1.57 ± 0.61	1.54 ± 0.65
*P*	>.05	>.05	>.05	<.05	<.05

No significant differences were observed in the dimensions of mobility, self-care, and usual activities between the before and posttreatment (*P* > .05). In contrast, there was a notable improvement in the dimensions of pain/discomfort and anxiety/depression following treatment as compared to the baseline measures (*P* < .05).

### 3.3. Adverse events

Other than occasional injection-related pain and minimal bleeding or bruising, which were rare, minor, and self-limited, no other adverse effects were observed. These side effects were temporary and fully reversible. No serious adverse effects were noted.

## 4. Discussion

Our study showed that D10W IH effectively decrease pain in the short and long term in the treatment of refractory MPS. After a cumulative 2 or 3 injections, long-term follow-up (12 weeks) showed satisfactory pain and anxiety/depression relief. There were no serious adverse effects after injection. To the best of our knowledge, this study represents the inaugural investigation assessing the efficacy of IH with D10W for MPS, with a focus on pain and anxiety/depression reduction and the incidence of adverse events.

The prevalence of myofascial pain is challenging to determine due to the absence of standardized diagnostic criteria. Reported prevalence estimates vary widely, with studies suggesting different values. However, the estimated overall prevalence stands at 46%.^[9]^ Management of MPS encompasses a variety of therapeutic approaches, such as physical therapy, acupuncture, massage, and dry needling. These interventions are efficacious in alleviating symptoms for a substantial proportion of affected individuals. Nonetheless, a subset of patients exhibits limited responsiveness to conventional therapeutic interventions. As an alternative to invasive therapies, IH has been widely applied in recent years, as fascial adhesion is considered a significant factor in the pathogenesis of MPS.^[10]^ The application of dextrose as a prolotherapy^[11]^ and nerve hydrodissection^[12]^ agent in the treatment of chronic musculoskeletal degeneration has gained significant traction in recent years. In the procedure of ultrasound-guided hydrodissection, clinicians utilize ultrasonographic guidance to administer a therapeutic solution into the interfascial layers, encompassing subcutaneous tissue, the epimysial compartment, the periosteal-fascial interface, and the tendon periphery, at the location where patients report the most intense pain.^[13]^ This targeted area is typically at the muscle periphery identified in the diagnosis of MPS.

Anatomically, the fascia is categorized into superficial and deep strata, which exhibits pronounced involvement in MPS. The fascia contains a dense sensory nerve endings closely related to the pain of MPS and may be associated with peripheral and central sensitization.^[14]^ Fascia coordinates muscle activity and forms a relatively sliding space between adjacent muscles, facilitating their relative movement. The fascial spaces are richly innervated and can transmit pain signals when there is harmful stimulation. Muscular overuse or injury can lead to changes in both the quantity and quality of hyaluronic acid within the fascial spaces, thereby increasing viscosity.^[15]^ These alterations result in diminished fascial glide, which in turn promotes adhesion. This adhesion can activate nociceptors located within the fascial space, consequently evoking the pain symptoms characteristic of MPS.^[16]^ Prolonged muscle contractions can increase metabolic stress and decrease blood circulation. This persistence undoubtedly intensifies the symptoms associated with MPS.^[17]^

**Figure 2.**
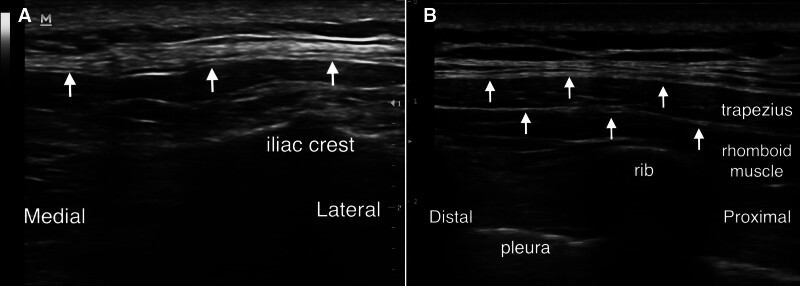
Ultrasound findings in patients with myofascial pain syndrome (MPS) reveal localized thickening and increased echogenicity of the fascia. (A) The white arrow points to the thickened, hyperechoic thoracolumbar fascia. (B) The white arrow indicates the thickened, hyperechoic fascia overlying the trapezius and rhomboid muscles. Both 2 patients experience significant localized pain.

The patients included in this study were those with MPS who had poor responses to conventional treatments. We hypothesize that the patients’ lack of response to conventional therapies is primarily attributed to fascial thickening and interfascial adhesion. Using elastographic ultrasound imaging, we observed that the fascia at the trigger points in these patients was significantly thicker and demonstrated increased stiffness (see Fig. 2). We hypothesize that the mechanism by which 10% dextrose IH can improve the pain of refractory MPS is (1) the immediate analgesic effect of dextrose on the sensory nerve endings in the fascia, (2) the accelerated flushing out of algogenic substance by the injected liquid, (3) dextrose provides energy to the nerve endings within the adherent fascial spaces, thereby alleviating the energy crisis; (4) the injected liquid facilitates the separation of adhesions within the myofascial spaces, thereby reducing friction during muscle contraction; (5) the appropriate hypertonic substance induces local inflammation and stimulates a proliferative cascade reaction, thereby repairing damaged tissue. The repair and remodeling of tissue are associated with long-term symptom relief in patients with chronic musculoskeletal pain.

The persistent presence of chronic neuropathic pain is intimately associated with the upregulation of the capsaicin receptor, known as transient receptor potential vanilloid receptor-1 (TRPV1).^[18]^ TRPV1 ion channels are predominantly found in 40% of the peripheral somatosensory nervous system, particularly within C-fiber neurons, which are responsible for the transmission of pain signals. Dextrose exerts an antagonistic influence on TRPV1, thereby directly contributing to analgesic effects.^[19]^ Furthermore, chronic neuropathic pain may be indicative of local hypoglycemia surrounding the affected nerve endings. The administration of dextrose can rapidly ameliorate this hypoglycemic condition, consequently alleviating neuropathic pain. It is also noteworthy that the distal branches of the dorsal rami of the spinal nerves course between the fascial layers of the trunk, which is particularly relevant in the context of hydrodissection treatment for the rhomboid and levator scapulae myofascial spaces. In this sense, hydrodissection releases the perineural adhesions and counteracts chronic neuropathic pain.^[20]^

Another possible reason for the long-term effect of IH is the regular self-stretching training performed by patients after injection. Fernandez suggests that comprehensive management of MPS should include self-management and exercise plans.^[21]^ IH improved pain and function in the short term, but regular fascial stretching training by patients enhanced the long-term effect of the injection treatment.

Although dextrose is widely used in nerve and fascial hydrodissection therapy, the optimal injection concentration and administration frequency have yet to be definitively established. Chou and colleagues used 15% dextrose in the treatment of 45 patients with chronic myofascial pain,^[22]^ while Kim in another study, found significant improvement in musculoskeletal symptoms after a single injection of 12.5% dextrose.^[23]^ 2 retrospective series, including 127 and 177 patients who received 20% dextrose injection treatment, showed definite relief from chronic myofascial pain and dysfunction.^[24,25]^ The dextrose concentrations used in these studies were inconsistent, and a consensus on the optimal concentration has not yet to be established. In our study, we used 10% dextrose. On one hand, 10% dextrose alleviates fascial adhesion by expanding the fluid space; on the other, it supplies energy to local nerve endings. Additionally, its slightly higher osmotic pressure compared to 5% isotonic dextrose exerts a prolotherapy therapeutic effect on damaged tissue, promoting repair. To minimize the potential adverse effects of hypertonicity on nerve endings, particularly near nerve trunks, we avoided using higher dextrose concentrations. Instead, we recommend isotonic 5% dextrose for injections in proximity to nerve trunks.

In our study, no significant differences were observed in the dimensions of mobility, self-care, and usual activities on the EQ-5D-5L questionnaire before and after treatment. This finding may correlate with the composition of our patient cohort, which included patients with MPS affecting the upper limbs, lower limbs, and trunk, and most of these patients did not present with substantial limitations in mobility, self-care, or usual activities. Conversely, posttreatment assessments revealed significant improvements in pain/discomfort, and anxiety/depression compared to baseline. This improvement is likely due to the inclusion of chronic MPS patients, whose persistent pain is closely linked to increased anxiety and depressive symptoms.

This study has several limitations. First, the sample size is relatively small. Second, its retrospective design limits the strength of the findings, and the absence of a control group prevents direct comparisons with other treatments. Third, although dextrose fascial hydrodissection has demonstrated clear clinical efficacy for refractory MPS, the optimal treatment parameters, including concentration, injection volume, and treatment course, remain undetermined. Future large-scale, controlled, prospective studies are needed to further evaluate the effects of IH in refractory MPS patients.

In summary, D10W IH effectively alleviates pain and anxiety/depression in patients with refractory MPS, providing both short-term and long-term relief. This therapeutic approach is considered feasible, safe, and cost-effective. Future randomized controlled clinical trials are necessary to assess the efficacy of IH in comparison to other treatment options for MPS patients.

## Acknowledgments

We extend our sincere acknowledge to our esteemed physical therapists, Li Yangzheng and Zhang Lingqi, for their significant contributions during the functional assessment for the patients and the data collection phase of the project.

## Author contributions

**Conceptualization:** Tao Wu, Bao Ru.

**Data curation:** Tao Wu.

**Formal analysis:** Haixin Song.

**Funding acquisition:** Tao Wu, Zhiping Liao, Xiaotian Yang.

**Investigation:** Haixin Song, Zhiping Liao, Fangchao Wu.

**Methodology:** Haixin Song, Zhiping Liao.

**Project administration:** Bao Ru.

**Software:** Xiaotian Yang, Fangchao Wu.

**Supervision:** Jianhua Li.

**Validation:** Zhiping Liao.

**Writing – original draft:** Tao Wu.

**Writing – review & editing:** Bao Ru, Jianhua Li.

## Supplementary Material


